# Integrated in silico assessment of the regulatory and structural consequences of pulmonary tuberculosis-associated *SP110* polymorphisms

**DOI:** 10.1038/s41598-026-65881-y

**Published:** 2026-08-06

**Authors:** Denis S. Kyabaggu, Alfred Ssekagiri, Eric Katagirya, Bernard S. Bagaya, Moses L. Joloba, Irene Andia-Biraro, David Patrick Kateete, Harriet Mayanja-Kizza, Lydia Nakiyingi

**Affiliations:** 1 Department of Immunology and Molecular Biology, School of Biomedical Sciences, Makerere University College of Health Sciences, Kampala, Uganda; 2 Department of Medical Microbiology, School of Biomedical Sciences, Makerere University College of Health Sciences, Kampala, Uganda; 3 Department of Medicine, School of Medicine, Makerere University College of Health Sciences, Kampala, Uganda; 4 Ministry of Health, Kampala, Uganda

**Keywords:** *SP110*, Single-nucleotide polymorphisms, Transcription factor binding, RNA secondary structure, *In silico* functional analysis, Immune regulation, Computational biology and bioinformatics, Diseases, Genetics

## Abstract

The molecular mechanisms underlying conflicting findings of association of *SP110* single nucleotide polymorphisms (SNPs) with susceptibility to pulmonary tuberculosis (PTB) remain unclear. We selected ten *SP110* SNPs from publicly available genomic databases, based on prior PTB association and minor allele frequencies exceeding 1% in African populations and identified in a Ugandan cohort with PTB. We then applied in silico multi-tool framework, incorporating deep-learning splice predictors including SpliceAI, Pangolin, and AlphaGenome, to evaluate the regulatory and functional consequences of the SNPs on transcription factor binding, RNA splicing, RNA secondary structure, and protein-level effects. The model captured population-relevant *SP110* variations rather than a directly genotyped Ugandan cohort. Our analysis revealed allele-specific gains and losses involving transcription factors TFAP2A, TFAP2C, TP63, Zfx, and IRF1, suggesting modulation of regulatory potential through altered transcription factor binding motifs. However, SpliceAI and Pangolin uniformly predicted low splice-disruptive effects across both exonic and intronic variants, while multiple protein pathogenicity tools consistently classified missense and stop-gained variants as benign or tolerated. RNA secondary structure analysis predicted that most variants preserved global thermodynamic stability. From our findings, *SP110* variants are unlikely to exert major effects through splicing disruption or protein destabilization but may contribute to functional diversity through subtle regulatory mechanisms, including transcription factor binding modulation and RNA structural reorganization.

**Supplementary Information:**

The online version contains supplementary material available at https://doi.org/10.1038/s41598-026-65881-y.

## Introduction

 The *SP110* gene produces a nuclear body protein that regulates transcription, organizes chromatin, and modulates immune responses, particularly within interferon-regulated pathways^1^. Multiple studies link *SP110* single nucleotide polymorphisms SNPs with susceptibility to tuberculosis, particularly pulmonary tuberculosis (PTB), highlighting their important role in host defense and immune regulation^2–7^. Prior studies have focused on *SP110* polymorphisms and population-level genetic associations, while relatively few have examined how *SP110* influences transcriptional control, particularly in PTB pathogenesis^8,9^. Transcription factors regulate gene expression by binding specific regulatory motifs in promoters, enhancers, and other regulatory elements^10^. Even single-nucleotide substitutions can markedly alter transcription factor binding affinity and, in turn, affect transcriptional output^11^. Accumulating evidence shows that SNP-induced changes in transcription factor binding reshape regulatory networks and contribute to the genetic architecture of complex and immune-mediated diseases, including PTB^11,12^. In the context of *SP110*, SNPs may modulate transcription factor binding, particularly for immune-relevant regulators such as Transcription Factor AP-2 Alpha/Gamma (TFAP2A/TFAP2C), Tumor protein P63 (TP63), Interferon Regulatory Factor 1 (IRF1), and Zinc Finger Protein X (Zfx), which could directly influence pathways governing cell differentiation, stress responses, and antimicrobial immunity^1^. *SP110* has attracted considerable attention because studies associate it with susceptibility to infectious and inflammatory diseases, including tuberculosis, and immune dysregulation disorders, highlighting its important role in host defense mechanisms^5,6,13–15^. Despite this biological importance, the regulatory impact of genetic variation within *SP110*, particularly on transcription factor binding, remains incompletely characterized in humans^12,15–19^.

Elucidating how regulatory *SP110* SNPs alter transcription factor binding affinity is essential for understanding gene content-specific expression and their potential contribution to immune-related phenotypes and disease susceptibility^20^. Computational (in silico) prediction tools that integrate positional weight matrices derived from experimentally validated transcription factor motifs effectively identify such regulatory disruptions, enabling allele-specific comparisons of transcription factor binding affinity^21–24^.

Platforms such as SNP2TFBS, SpliceAI, and Pangolin enable high-throughput identification of SNPs that may weaken or strengthen transcription factor-DNA interactions, thereby helping researchers prioritize candidate regulatory variants for functional validation^21,25,26^. When applied rigorously, in silico transcription factor binding site analyses may offer cost-effective and reproducible approaches for refining genotype-phenotype hypotheses and guiding downstream molecular studies.

Our study, therefore, set out to conduct an integrative in silico analysis of *SP110* SNPs with prior PTB association and minor allele frequency > 1% in African populations, and with specific epidemiological relevance to a Ugandan cohort affected by pulmonary tuberculosis (PTB), to evaluate their potential role in modulating transcription factor binding and RNA secondary structure conformation as a basis for the prediction of underlying functional mechanisms of association with PTB.

## Materials and methods

### Study design

We applied an in silico analytical framework (*Supplementary table*,* S1*) to evaluate the functional effects of 10 *SP110* SNPs (*rs9061*,* rs11556887*,* rs7580900*,* rs7580912*,* rs2241525*,* rs3948464*,* rs2114592*,* rs6436915*,* rs1427294*, and *rs1346311*) on gene regulation and post-transcriptional processes. Variants were selected based on prior association with PTB and minor allele frequency > 1% in African populations, together with specific biological and epidemiological relevance to a Ugandan cohort with PTB. Specifically, the selection process incorporated evidence from prior association studies linking *SP110* variation to tuberculosis^6,7,13^ and related immune-mediated conditions^14^, alongside data from publicly available reference and annotation resources, including the NCBI dbSNP database^27^, the human reference genome GRCh38.p14 (NCBI Assembly: NC_000002.12), and JASPAR CORE transcription factor binding profiles^22^. These resources were used strictly as reference datasets for annotation, motif comparison, and contextual genomic interpretation rather than as training data for model development. No primary dataset was generated for this study, and no machine learning or statistical models were trained on any proprietary, newly generated, or study-specific dataset. The population process also considered linkage equilibrium structure across the *SP110* locus to capture representative genetic variation while minimizing redundancy. Consistently, the final variants set included both previously reported disease-associated loci and common polymorphisms with potential regulatory significance. Reference to a Ugandan pulmonary tuberculosis context reflects the epidemiological framework guiding variant prioritization rather than a directly sampled cohort. All analyses used genomic coordinates derived from the GRCh38.p14 human genome assembly (NCBI accession: NC 000002.12) to ensure consistency across computational tools Table 1.

**Table 1:** Summary of *SP110* SNPs analysed in this study.

rsID	Chromosome	GRCh38 coordinate	Reference allele	Alternate allele	Variant Consequence	Transcript ID	Source
rs11556887	Chr 2	2:230212961	G	A	Missense	ENST00000258381.11	dbSNP/Ensembl VEP
rs1346311	Chr 2	2:230217672	G	A	Intron	ENST00000258381.11	dbSNP/Ensembl VEP
rs1427294	Chr 2	2:230213733	G	A	Intron	ENST00000258381.11	dbSNP/Ensembl VEP
rs2114592	Chr 2	2:230210491	G	A	Intron	ENST00000258381.11	dbSNP/Ensembl VEP
rs2241525	Chr 2	2:230178086	C	T	Intron	ENST00000258381.11	dbSNP/Ensembl VEP
rs3948464	Chr 2	2:230185999	A	C	Missense	ENST00000258381.11	dbSNP/Ensembl VEP
			A	G	Missense	ENST00000258381.11	dbSNP/Ensembl VEP
			A	T	Stop-gained	ENST00000258381.11	dbSNP/Ensembl VEP
rs6436915	Chr 2	2:230169808	G	A	Intron	ENST00000258381.11	dbSNP/Ensembl VEP
rs7580900	Chr 2	2:230216669	T	A	Intron	ENST00000258381.11	dbSNP/Ensembl VEP
			T	C	Intron	ENST00000258381.11	dbSNP/Ensembl VEP
rs7580912	Chr 2	2:230216690	T	C	Intron	ENST00000258381.11	dbSNP/Ensembl VEP
rs9061	Chr 2	2:230212395	C	T	Missense	ENST00000258381.11	dbSNP/Ensembl VEP

### Prediction of pre-mRNA splicing disruptions

We assessed the splicing effects of SP110 variants using SpliceAI. SpliceAI generates scores that range from 0 to 1, interpreted as the probability that a variant affects splicing within a ± 500 bp window. These scores are reported for four kinds of splicing alterations: acceptor gain, acceptor loss, donor gain, and donor loss. Following Jaganathan et al. (2019)^26^, cutoffs of 0.2 (high recall), 0.5 (recommended), and 0.8 (high precision) were applied. The reported position offsets correspond to the position of greatest change in probability within the window, with negative values indicating positions to the left of the variant in genomic coordinates, regardless of transcript strand. SpliceAI outputs were further interpreted using SAI-10k-calc to classify the type of splicing aberration (pseudoexonization, whole or partial intron retention, partial exon deletion, or exon skipping), the size of the inserted or deleted sequence, and the effect on reading frame^28^. Canson et al. (2023)^28^ established SpliceAI delta score thresholds for this classification: 0.02–0.2 for exon skipping or whole-intron retention, and 0.02–0.05 for pseudoexon gain, with a gain-of-exon size range of 25–500 bp. Splicing predictions were independently cross-checked using Pangolin, which also generates delta scores (0–1) within a ± 500 bp window^25^.

### Assessment of protein-level effects of missense variants

We used multiple tools to assess protein-level functional consequences. These included REVEL, AlphaMissense, PolyPhen-2, SIFT, and PrimateAI-3D. Variants were classified using calibrated thresholds developed by Pejaver et al. (2022)^29^ and Bergquist et al. (2025)^30^. Each score is assigned to a graded strength-of-evidence category with a corresponding point value. For AlphaMissense, scores ≥ 0.990 were classified as Strong Pathogenic (+ 4), 0.972–0.989 as Pathogenic (+ 3), 0.906–0.971 as Moderate Pathogenic (+ 2), and 0.792–0.905 as Supporting Pathogenic (+ 1); scores ≤ 0.070 were classified as Benign (− 3), 0.071–0.099 as Moderate Benign (− 2), and 0.100-0.169 as Supporting Benign (− 1), with 0.170–0.791 considered Indeterminate (0), as calibrated by Bergquist et al. (2024). For REVEL, scores ≥ 0.932 were classified as Strong Pathogenic (+ 4), 0.879–0.931 as Pathogenic (+ 3), 0.773–0.878 as Moderate Pathogenic (+ 2), and 0.644–0.772 as Supporting Pathogenic (+ 1); scores ≤ 0.016 were classified as Strong Benign (− 4), 0.017–0.052 as Benign (− 3), 0.053–0.183 as Moderate Benign (− 2), and 0.184–0.290 as Supporting Benign (− 1), with 0.291–0.643 considered Indeterminate (0), as calibrated by Bergquist et al. (2025)^30^. SIFT, PolyPhen-2, and PrimateAI-3D did not reach Strong-level evidence under the original Pejaver et al. (2022)^29^ calibration and were classified only up to the Moderate level: for SIFT, scores of 0-0.001 corresponded to Supporting Pathogenic (+ 1) and a score of 0 to Moderate Pathogenic (+ 2), while scores of 0.080–0.327 corresponded to Supporting Benign (− 1) and scores ≥ 0.327 to Moderate Benign (− 2); for PolyPhen-2, scores of 0.978–0.999 corresponded to Supporting Pathogenic (+ 1) and scores ≥ 0.999 to Moderate Pathogenic (+ 2), while scores of 0.009–0.113 corresponded to Supporting Benign (− 1) and scores ≤ 0.009 to Moderate Benign (− 2); for PrimateAI, scores of 0.790–0.867 corresponded to Supporting Pathogenic (+ 1) and scores ≥ 0.867 to Moderate Pathogenic (+ 2), while scores of 0.362–0.483 corresponded to Supporting Benign (− 1) and scores ≤ 0.362 to Moderate Benign (− 2).

### Modeling of local mRNA secondary structure

The effect of each SNP on local mRNA secondary structure was modeled using the RNAfold web server from the Vienna RNA WebSuite^31^. For each variant, the analysis extracted genomic sequences spanning ± 160 nucleotides centered on the SNP position directly from the NCBI SNP Database via the VarView interface by querying individual rsIDs^31,32^. This window size allowed sufficient sequence context to capture local structural changes without introducing distal noise.

The analysis used reference and alternate allele sequences, obtained by substituting corresponding nucleotides using Unipro UGENE version 48.0^33^. RNAfold computed Minimum Free Energy (MFE) values for both optimal and centroid structures under default thermodynamic parameters^31^. We quantified structural impact as the difference in MFE (∆MFE) between reference and mutant sequences, with larger absolute ∆MFE values indicating greater perturbations of RNA stability.

### Analysis of transcription factor binding site alterations

We evaluated transcription factor binding site alterations using SNP2TFBS^21^. It generates allele-specific transcription factor binding predictions using position weight matrices (PWMs) derived from the JASPAR CORE 2020 vertebrate database^22^. SNP2TFBS takes variant identifiers as input for each SNP and computes binding scores as the sum of the position-specific weights over all bases of the binding site, separately for the reference and alternate alleles. The difference in predicted binding affinity (ScoreDiff) is calculated as:

$$ScoreDiff_{~} = \Sigma _{{i = 1}} ^{L} w_{{i,b_{i}^{{alt}} }} ~ - ~\Sigma _{{i = 1}} ^{L} w_{{i,b_{i}^{{ref}} }}$$

where *L* is the motif length, *w*_*i, b*_ is the PWM weight assigned to nucleotide *b* at position *i*, and *b*_*i*_^*ref*^ and *b*_*i*_^*alt*^ denote the reference and alternate nucleotides, respectively.

A positive ScoreDiff (> 0) indicates that the alternate allele has a higher PWM binding score than the reference allele, meaning the genetic variation likely creates or strengthens a transcription factor binding site (TFBS). Conversely, a negative ScoreDiff (< 0) shows that the alternate allele has a lower PWM binding score than the reference allele, indicating that the variation likely disrupts or weakens an existing TFBS. A zero ScoreDiff (= 0) signifies that both alleles yield identical raw binding scores, meaning the SNP does not alter the predicted binding strength for that transcription factor.

### Cross-modality regulatory effect prediction

We assessed the predicted regulatory effects of the 10 SP110 variants using AlphaGenome (Google DeepMind), a unified DNA sequence model that predicts genome-wide functional tracks from 1 Mb input sequences at single-base resolution. Histone modification and transcription factor binding effects were quantified using the CenterMaskScorer, which computes the log2 fold-change between alternate and reference predicted signal within a defined window centered on the variant. Tracks were queried across biosamples relevant to pulmonary tuberculosis pathophysiology, including bronchial epithelial cells, lung fibroblasts, and multiple lung lobe/tissue segments, spanning ten histone marks (H3K27ac, H3K27me3, H3K36me3, H3K4me1, H3K4me2, H3K4me3, H3K79me2, H3K9ac, H3K9me3, H2AFZ) and four transcription factor/RNA polymerase tracks (CTCF, EP300, POLR2A, POLR2AphosphoS5). Splicing effects were assessed using AlphaGenome’s splice-junction, splice-site, and splice-site-usage output heads, with an aggregate splicing score computed as a composite summary across the three tracks per variant.

### Integration of functional predictions

Our analysis integrated predictions from splicing, protein function, RNA structure, and transcription factor binding to generate a comprehensive functional profile for each *SP110* SNP. This multi-layered approach enabled the identification of variants with converging evidence of regulatory or functional impact. Variants showing consistent effects across multiple analytical domains were prioritized as candidate regulatory SNPs. This integrative framework strengthened biological interpretation and provided a rational basis for selecting variants for future experimental validation.

### Computing infrastructure

All web-based analyses were performed on a personal computer running *Windows 10/11* or *macOS Tahoe version 26.4.1* using *Google Chrome version 147.0.7727.56* (Official Build, x86_64). Sequence editing before RNAfold submission was performed using Unipro UGENE version 48.0 on *macOS Tahoe version 26.4.1*. Web-based tools were accessed between July 2025 and December 2025; access dates for each tool are reported in *Supplementary Table **S1*. No high-performance computing infrastructure was required.

### Data integrity

Data integrity was verified before analysis. All 10 *SP110* SNPs were confirmed as valid dbSNP entries by querying their rsIDs against NCBI dbSNP Build 156 and cross-referencing genomic coordinates with the GRCh38.p14 reference assembly (NC_000002.12). Variant consequence annotations were verified against Ensembl VEP and dbSNP entries. Sequence windows extracted via NCBI VarView were visually inspected to confirm correct centering on the variant position and accurate reference/alternate allele representation before submission to analytical tools.

### Patient and public involvement

It was not possible to involve patients or the public in the design, conduct, reporting, or dissemination plans for our research because our study methods were predictive in nature and utilised in silico (computational) tools not widely available in the public domain in Uganda.

### Ethical approval and consent to participate

Although the present analyses were conducted solely on publicly available data and would not independently require ethical approval or informed consent under international research ethics guidelines, the study forms part of a broader research framework that received ethical clearance from the Uganda National Council for Science and Technology (SS870ES). The study methods and experimental protocols were approved by the Makerere University College of Health Sciences School of Biomedical Sciences Institutional Review Board (Approval SBS-744) in accordance with the Declaration of Helsinki and relevant institutional guidelines and regulations. Informed consent was obtained for the primary data collection within that broader study. The current in silico component did not involve access to or use of participant-level data from that cohort. Throughout the study, results were interpreted and reported responsibly, with careful emphasis that all findings represent computational predictions rather than experimentally or clinically validated effects, thereby avoiding overstatement of their biological or clinical significance.

## Results

### Allele-specific effects of* SP110* variants on transcription factor binding

Out of ten SNPs analyzed using the SNP2TFBS, only four returned high-confidence predicted transcription factor binding site alterations. For the intron 1 variant *rs1346311* (G > A), the alternate allele was predicted to disrupt binding affinity for both TFAP2A (ScoreDiff = -82) and TFAP2C (ScoreDiff = -207). Conversely, the intron 1 variant r*s2114592* (G > A) showed no predicted change in binding for ZNF354C (ScoreDiff = 0). The third intron 1 variant, *rs7580900* (T > C), showed opposing, factor-specific effects: the alternate allele is predicted to disrupt binding for transcription factor TP63 (ScoreDiff = -242) while simultaneously enhancing the binding affinity for Zfx (ScoreDiff = 118). Finally, the exonic variant rs9061 (C > T) was predicted to enhance the binding affinity for IRF (ScoreDiff = 307).

### In silico analysis of* SP 110* variants on splicing and protein function

Three missense variants (*rs11556887*, *rs3948464*, and *rs9061*) and one stop-gained allele of *rs3948464* were evaluated using multiple in silico prediction tools (Table 2). SpliceAI predicted generally low splice-altering effects, with maximum Δ scores ranging from 0.01 to 0.21. The highest predicted splice alteration was observed for the *rs3948464* A > T stop-gained allele (maximum Δ = 0.21), whereas *rs11556887* and r*s3948464* A > C each had a maximum Δ score of 0.03, *rs3948464* A > G had a score of 0.02, and *rs9061* had the lowest score (0.01). Predicted splice changes involved acceptor and donor gain or loss events occurring within 5–200 bp of the variant position. Pangolin similarly predicted minimal splice disruption, with splice disruption probabilities ranging from 0.00 to 0.14 for splice loss and from 0.00 to 0.01 for splice gain.

At the protein level, the missense alleles resulted in single amino acid substitutions, including Leu > Trp for rs3948464 A > C, Leu > Ser for rs3948464 A > G, and Glu > Lys for rs9061. In contrast, the rs3948464 A > T allele introduced a premature termination codon and was predicted to result in the deletion of a 50-amino-acid sequence, reducing the predicted protein length from 713 to 663 amino acids.

Functional prediction scores for the missense variants were generally low, with PrimateAI-3D scores ranging from 0.01 to 0.44, REVEL scores from 0.035 to 0.083, and AlphaMissense scores from 0.0672 to 0.1191. PolyPhen-2 classified *rs11556887* as probably damaging (0.96), whereas *rs3948464* A > C (0.22), *rs3948464* A > G (0.00), and *rs9061* (0.007) were predicted to be benign. Similarly, SIFT predicted *rs11556887* to be at the threshold of deleteriousness (0.05), while rs3948464 A > C (0.19), rs3948464 A > G (0.47), and *rs9061* (0.29) were predicted to be tolerated.

**Table 2:** In silico splicing disruption predictions for SP110 SNPs.

rsID	Position (GRCh38)	Variant consequence	Max SpliceAI Δ	Δ type (position)	Pangolin SDP (Loss/Gain)	PrimateAI-3D	REVEL	Alpha Missense	PolyPhen-2	SIFT
rs11556887	230212961G > A	Missense	0.03	Acc loss (43 bp)	0.11 / 0.01	0.08	0.062	0.1191	0.96	0.05
			0.02	Don loss (-200 bp)						
			0.01	Acc gain (-182 bp)						
rs3948464	230,185,999 A > C	Missense	0.03	Acc loss (+ 144 bp)	0.01 / 0.00	0.44	0.083	0.1036	0.22	0.19
			0.02	Don loss (-5 bp)						
	230,185,999 A > G	Missense	0.02	Acc gain (+ 144 bp)	0.00/0.000	0.01	0.035	0.0743	0	0.47
			0.02	Don gain (-5 bp)						
	230,185,999 A > T	Stop-gained	0.21	Acc loss (+ 144 bp)	0.14/0.01	--	--	--	--	--
			0.15	Don loss (-5 bp)						
rs9061	230,212,395 C > T	Missense	0.01	Don loss (-48 bp)	0.02 / 0.01	0.05	0.053	0.0672	0.007 (benign)	0.29 (tol.)

### *SP110* variants and varied predicted immune responses

AlphaGenome ChIP-seq predictions across the 10 *SP110* variants showed effects on histone marks in lung-relevant biosamples (bronchial epithelial cells, lung fibroblasts, and multiple lung lobes/tissue segments), with most variants producing a top-ranked quantile score exceeding ± 0.90. The strongest effects were observed for chr2:230216690:T > C, which increased predicted H3K27ac signal in left lung tissue (quantile score = 0.9998), and chr2:230185999:A > T, which produced the largest negative effect, decreasing H3K36me3 signal in the lower lobe of the right lung (quantile score = -0.9994). H3K36me3 and H3K4me3 marks were the most frequently disrupted marks.

Across the transcription factor tracks, CTCF was the predominant TF affected, ranking as the top hit for 11 of 13 variants, with quantile score magnitudes ranging from 0.57 to 0.99 across bronchial epithelial cells and multiple lung tissue biosamples. The largest CTCF disruptions were seen for chr2:230216669:T > A (quantile = -0.9885) and chr2:230216669:T > C (quantile = 0.9759), both in bronchial epithelial cells. Notably, two variants departed from this pattern: chr2:230216690:T > C showed its strongest effect on RNA polymerase II phosphorylated at Ser5 (POLR2AphosphoS5; quantile = 0.9993, upper lobe of left lung), and chr2:230210491:G > A showed its top effect on unphosphorylated POLR2A (quantile = -0.5674).

Predicted splicing effects were considerably more variant-specific than the histone or TF findings, with only one variant, chr2:230185999:A > T, showing an aggregate AlphaGenome splicing score of 0.465, driven predominantly by a strong effect on the splice-junction track (1.663) rather than splice-site strength or site-usage tracks. Two additional variants, chr2:230212961:G > A and chr2:230213733:G > A, showed moderate splicing scores (0.356 and 0.210, respectively), both similarly driven by elevated splice-junction scores relative to splice-site and site-usage components. The remaining ten variants produced aggregate splicing scores below 0.15.

### Variant-specific effects of* SP110* polymorphisms on RNA secondary structure stability

The predicted effects of the ten SNPs on mRNA secondary structure are summarized in Supplementary Table S1. Overall, most variants had minimal effects on the minimum free energy (MFE) of the predicted RNA structure, with absolute MFE differences (|ΔMFE|) ranging from 0.00 to 4.70 kcal/mol. The largest change in MFE was observed for *rs6436915* (|ΔMFE| = 4.70 kcal/mol), followed by *rs3948464* A > C (2.50 kcal/mol), whereas rs1346311 and *rs7580912* each showed moderate changes of 1.40 kcal/mol, *rs7580900* T > A showed a difference of 1.20 kcal/mol, and *rs2241525* and *rs9061* showed smaller changes of 0.70 and 0.50 kcal/mol, respectively. Variant *rs1427294* showed an MFE difference (0.10 kcal/mol), while *rs11556887* and *rs2114592* showed no change in MFE between the reference and variant sequences (|ΔMFE| = 0.00 kcal/mol).

Greater variation was observed in the centroid structures, with absolute centroid energy differences (|ΔCentroid|) ranging from 0.10 to 47.70 kcal/mol. The largest centroid energy change was predicted for *rs6436915* (47.70 kcal/mol), followed by *rs2241525* (25.77 kcal/mol), *rs3948464* A > C (9.77 kcal/mol), rs11556887 (6.39 kcal/mol), and *rs9061* (6.13 kcal/mol). Smaller centroid energy differences were observed for *rs1346311* (2.70 kcal/mol), *rs7580900* T > A and *rs7580912* (1.20 and 1.40 kcal/mol, respectively), *rs2114592* (0.50 kcal/mol), and *rs1427294* (0.10 kcal/mol). Comparison of the MFE and centroid structures (*Supplementary Fig. 1*) showed that several variants, particularly *rs6436915*, *rs2241525*, *rs3948464*, *rs11556887*, and *rs9061*, produced substantially larger changes in centroid energy than in MFE.

## Discussion

### Implications of allele-specific modulation of* SP110* transcriptional regulation by single nucleotide polymorphisms

Our findings suggest particular *SP110* variants modulate gene regulation by altering the transcription factor binding landscape. The intronic *rs1346311* is predicted to reduce binding affinity of TFAP2A and TFAP2C, mirroring previous studies which showed that AP-2 transcription factors play key roles in regulating immune- and inflammation-related genes^34^. Earlier studies showed intronic variants can disrupt enhancer or silencer elements, leading to diminished transcriptional activation without altering coding regions^35,36^. The predicted loss of TFAP2 binding motifs reinforces previous evidence linking reduced AP-2 activity to regulatory dysregulation in immune pathways^37^.

In contrast, the absence of detectable transcription factor binding changes associated with *rs2114592* suggests that not all intronic *SP110* polymorphisms exert functional regulatory effects. This observation aligns with genome-wide functional annotation studies indicating that a substantial proportion of intron variants remain selectively neutral with respect to transcription factor binding and gene regulation^36,38^. It has been argued that the regulatory consequences of intronic SNPs are highly context-dependent, reinforcing the importance of variant-specific functional screening rather than assuming uniform regulatory impact across intron regions^39^.

The divergent effects of *rs7580900* on TP63 and Zfx binding may reflect a regulatory trade-off that has been widely reported in transcriptional network studies, with prior research demonstrating that single-nucleotide changes can reshape transcription factor occupancy by redistributing binding preferences rather than simply abolishing regulatory interactions^40^. TP63 regulates cellular differentiation and mediates stress responses, while Zfx maintains transcriptional programs and supports cell survival, particularly in immune and stem cell populations^36^. The opposing predicted binding effects observed at this locus, therefore, suggest *rs7580900* may redirect *SP110* regulation toward alternative transcriptional programs.

The exon variant *rs9061* predicted increased IRF1 binding, supporting earlier evidence that exonic SNPs can regulate transcription independently of amino acid substitutions^41^. Previous studies have established IRF1 as a key regulator of immune and inflammatory gene expression, with enhanced IRF1 binding linked to increased transcriptional responsiveness during infectious and inflammatory conditions^42,43^. Previous studies also show that exonic variants modify transcription factor recruitment through local DNA sequence changes rather than altering protein structure^44,45^. Taken together, our findings suggest *rs9061* may enhance *SP110* transcriptional regulation through a gain-of-function regulatory mechanism. By demonstrating predicted allele-specific gains and losses in transcription factor binding motifs, this study provides additional in silico support for a role of *SP110* genetic variation in immune regulation and disease susceptibility.

### *SP110* variants predominantly influence gene regulation rather than splicing or protein function

The present findings suggest *SP110* variants are unlikely to exert major functional effects through splicing disruption or protein destabilization, emphasizing the regulatory subtlety of genetic variation within this locus. The uniformly low SpliceAI ∆ scores across both exonic and intronic variants may indicate that these SNPs do not substantially disrupt canonical splice donor or acceptor sites. The missense variants *rs11556887* and *rs9061* exhibited concordantly benign predictions across multiple orthogonal tools, including PrimateAI-3D, REVEL, AlphaMissense, PolyPhen-2, and SIFT. This convergence aligns with a previous study showing that consensus benign classifications across diverse predictors strongly reduce the likelihood of pathogenic protein effects^46^. The pattern is especially evident in immune-related genes that tolerate moderate amino acid variability^47^. The weak splicing signals associated with these variants further support the interpretation that, if any, their functional relevance does not arise from altered exon-intron architecture.

Interestingly, the stop-gained variant *rs3948464 A > T*, was also predicted to have minimal functional impact by both splicing and protein pathogenicity models. Previous studies show that premature stop codons in alternatively spliced regions, or near the C-terminus, can evade nonsense-mediated decay^48^. Functional redundancy can further mitigate their phenotypic consequences^48,49^. This finding highlights the limitation of relying solely on variant consequence annotations without integrating quantitative functional predictions.

Multiple intronic variants, including *rs1346311* and *rs2114592*, showed no predicted splicing effects. These results support the assertion that intronic variants exert subtle regulatory effects rather than causing overt splicing disruption, often modulating transcription factor binding, a mechanism increasingly implicated in immune gene regulation^50^.

Collectively, our findings suggest that the functional effects of the analyzed *SP110* variants are unlikely to result from protein damage or splice-site disruption. Instead, when interpreted alongside transcription factor binding analyses reported in earlier studies, the data supports a model in which *SP110* SNPs influence gene function primarily through regulatory fine-tuning rather than structural perturbation. Prior genetic studies consistently show that *SP110* variation lacks strong evidence for high-penetrance coding mutations^51^.

### Implications of RNA secondary structure alterations as a post-transcriptional regulatory mechanism of* SP110* variants

We postulate that *SP110* SNPs may influence post-transcriptional regulation by altering RNA secondary structure stability rather than through widespread disruption of global folding energetics. Variants such as *rs11556887* and *rs9061* showed minimal changes in minimum free energy but moderated centroid shifts. Such localized structural rearrangements potentially affect RNA-protein interactions and transcript processing efficiency.

The pronounced centroid destabilization observed for intronic variants *rs2241525* and *rs6436915* is particularly notable. The large centroid shifts observed for these variants suggest altered structural conformations that may influence regulatory element accessibility, even in the absence of strong splice-site disruption signals. This finding aligns with previous reports indicating that RNA structural perturbations often act independently of canonical splicing predictors^52^.

The relatively modest minimum free energy changes observed across most variants are consistent with genome-wide analyses showing that disease-associated and regulatory SNPs frequently preserve overall RNA stability while reshaping structural conformational landscapes^53^. The stop-gained variant *rs3948464* further illustrates this principle, as its moderate centroid destabilization suggests potential effects on RNA surveillance or decay pathways rather than simple transcript truncation, a mechanism reported for premature termination variants that evade nonsense-mediated decay^48,54^.

Integrating predictions across the three regulatory modalities reveals two distinct functional signatures within this *SP110* region. The position chr2:230185999 (*rs3948464)* demonstrated the most consistently disruptive site in the dataset: all three possible substitutions at this base ranked among the strongest predicted CTCF-binding disruptions and showed extreme H3K36me3 effects, while the A > T allele specifically carried the highest splicing score of the entire variant set (*Supplementary data S3*). A second, distinct pattern is seen at the neighboring chr2:230216669–230,216,690 cluster (*rs7580900*), where the dominant predicted effect shifts away from CTCF toward active chromatin marks (H3K27ac, H3K4me3) and RNA Pol II engagement (POLR2AphosphoS5), consistent with a promoter- or enhancer-proximal element whose disruption is more likely to alter *SP110* transcriptional output than pre-mRNA splicing. The dominance of CTCF as the top transcription factor hit further suggests that this region of *SP110* harbors one or more architectural CTCF binding sites, disruption of which could plausibly influence chromatin looping or insulator function relevant to immune gene regulation in the context of pulmonary tuberculosis susceptibility. As these findings derive from sequence-based computational predictions rather than direct chromatin measurements, they should be regarded as variant-prioritization evidence; where feasible, experimental validation would strengthen any regulatory claims made in the manuscript.

Collectively, our findings support a growing body of evidence that alterations in RNA secondary structure may represent an underappreciated mechanism by which genetic variation modulates gene regulation. In the context of *SP110*, the observed variant-specific centroid shifts suggest RNA-level regulatory fine-tuning may complement transcription factor binding and chromatin-mediated mechanisms previously reported for this gene. This multi-layer regulatory model aligns with earlier studies which linked *SP110* polymorphisms to immune phenotypes but found little evidence of highly penetrant coding mutations^1^.

### Study limitations

Despite its integrative design, this study has limitations. All analyses relied on in silico prediction tools, which, although state-of-the-art, cannot fully capture the complexity of in vivo regulatory environments or cell-type-specific effects. Second, transcription factor binding predictions did not fully account for epigenetic modifications or three-dimensional genome organization, all of which may influence regulatory outcomes. To complement the framework used in this study, future studies may incorporate tools, such as DeepMEP, a deep learning post-translational prediction tool that may measure missense mutation effects on phosphorylation at a particular site based on its flanking sequences^55^. Third, RNA secondary-structure analyses assessed thermodynamic and centroid changes but did not directly model interactions with RNA-binding proteins or the splicing machinery. Finally, the absence of population-specific functional validation limits inference regarding the clinical or phenotypic relevance of these variants across diverse genetic backgrounds.

## Conclusion

Collectively, our study provides integrated in silico evidence that selected *SP110* single-nucleotide polymorphisms exert their functional influence predominantly through regulatory mechanisms rather than through overt disruption of splicing or protein structure. Across multiple complementary prediction frameworks, the analyzed variants showed uniformly low splicing disruption signals and consistently benign protein pathogenicity classifications, indicating a limited likelihood of deleterious effects on transcript architecture or protein stability. In contrast, allele-specific alterations in transcription factor binding affinity and pronounced variant-dependent changes in RNA secondary structure centroid conformations highlight regulatory fine-tuning as a principal mechanism of *SP110* functional modulation. These findings support a multilayered regulatory model in which *SP110* genetic variation shapes gene expression through subtle but potentially consequential effects on transcriptional regulation and post-transcriptional RNA dynamics, providing a mechanistic basis for previously reported associations between *SP110* polymorphisms and immune-related phenotypes. Based on the findings, we recommend that functional interpretation of *SP110* variants prioritize regulatory annotation over coding consequence classification alone. Integrative pipelines combining transcription factor binding prediction, RNA structural modeling, and splicing assessment should be adopted when evaluating intronic and weakly deleterious variants in immune-related genes. We further recommend caution in interpreting truncating or missense annotations without quantitative functional evidence, as several variants traditionally classified as high-impact showed minimal predicted functional consequences in this study.

Future studies should experimentally validate the predicted regulatory effects using cell-based assays, including electrophoretic mobility shift assays, reporter gene assays, and chromatin immunoprecipitation sequencing, to confirm allele-specific transcription factor binding. Additionally, subsequent studies should employ RNA structure probing methods and RNA-binding protein interaction assays to clarify the biological significance of the observed RNA centroid structure alterations. The studies should extend functional analyses across immune-relevant cell types and disease contexts to establish tissue-specific and condition-dependent regulatory effects. Future work should also integrate population genetics, expression quantitative trait locus analyses, and clinical phenotype data to translate these regulatory findings into a clearer understanding of *SP110-*associated immune susceptibility and disease risk.

## Supplementary Information

Below is the link to the electronic supplementary material.

Supplementary Material 1

Supplementary Material 2

## Data Availability

The datasets analysed during the current study were obtained from publicly available resources. SNP data are available in the NCBI dbSNP repository (https://www.ncbi.nlm.nih.gov/snp/) under the following rs accession numbers: rs11556887, rs1346311, rs1427294, rs2114592, rs2241525, rs3948464, rs6436915, rs7580900, rs7580912, and rs9061. SNP coordinates are reported relative to the GRCh38 human reference genome (RefSeq accession NC_000002.12). Sequence segments derived from the GRCh38 reference genome and used for RNA structure prediction are available at https://doi.org/10.5281/zenodo.19652998.
