# Supplementary material for: Integrated in silico assessment of the regulatory and structural consequences of pulmonary tuberculosis-associated SP110 polymorphisms
Source: Sci Rep. 2026 Aug 6;16:36800. doi: 10.1038/s41598-026-65881-y (PMC13736213; doi:10.1038/s41598-026-65881-y)
Supplement: Supplementary file 1 [file 41598_2026_65881_MOESM1_ESM.docx]

*rs1427294* RNA secondary	structure

*rs1346311* RNA secondary structure

T

A T A T A A

T T C

T G

G

A

T

G C A G T

C

A C A T T

T

G

G T T A C

C T T A T

G T G

T T

T T

A A

ACACCC A T G	C

G

T A

T

A T G T G T T C G G T A A

A A A

A G G G	T

A

A T

G C

TG T A T G

T

G

G A T T

T

T

C

A

A T

T G T

A A G

T A C C

C G TC GG C

C A G C

G T

A

G T A

A A

G

A G C

A

G T C

A T CC GT

CT T A

A C

C A

A C

C

C C

C T A CT T

G C G A AA

AC G C

T G T

A C

A T G

T

A C

T

GG

C

T C C T

T

A C C G T

A

A

C G

G

C T

A C G

G

C T

C G C G C G C G A T C G

T C

T C G T

T G

T

A

A	A

G T C A T

T	A

A A T G

T A G C C G C G

T A A T A

G CA

G T

A

T

T

C G G A

G T G CT

T G

G T G C G C

T G

T G

T A G

T

C	C

C G

T A

T

G

G

T T

T A

C G A C GC A	C

A C T

C

A A

T	A

T	C

C	T

T G C T

G C C G C G T A

C G

T

G T

T A G

C G A T

C G T G

A

A G

C GAT

T

G T T G

C G T A

G T

A T G C

CA T

A

C A

T T G

T T

T

T T

T C

T T T

A T

G C A

G T C

A	A

C	A G

T

C T

A G C T A T AA T

C

A C

G

T A

A T G T T

A

T G G

C C

T	AT G A T

T AC

T A G A C

C

T

C

G A A A C C A

C

C GTTCAA

C

T

T A

C C

T G A C

A

T A G C

C

A C A

AC G A A G G

T

CC G

T A

C C T AC T

T C

C A T G

C

C G T C G G

C T

G

A A

C T A C G

GC

T

C G

C G

C G

C T

A G

C G T T

T

C

A

T C T

G T

G

A T A A T

G T

G G G

T

A C

A

T G G T A T

A T

C G

T T C T A A T

G A

G A

A

A T

C T T

A

G

0

1

TG T

C G C G A T G C

G C

G C

G G T A

C G

G

C C T C G

T A

C G

A T C

A

A C

T

*rs2241525* RNA secondary structure

*rs3948464C/T* RNA secondary structure

*rs2114592,* RNA secondary structure

T C C T C

T T

C

T

G

G C

C

C A

T

C C TT T

T G

T

C T A	A

C

CCC A

G T

T

C

T C T

CG T G CA

C

AC

A G A T

T T T

T A

A

A

C T A T T

G A GC

C TA CT

G G C	C T	T

A C C A A C

A C A T T T

C

T A T C T A

T

C C A

T

C

T A	G G

G T C

C T A

G

T C C C A

T A

C T

G T G A

C T

C A T	G C C A G T

C A

G A T

T T

A C C A C C C G TACC

G G

C A

T T

C	C

T AC

T	G

T CG G G C A	T A G G TCT

C	C

G

T

T C C

T

C A T T T T CT T C T T C C A G G T T G A G T C G

G A G G T T C A A C T

A G T

T

T

A A C C T G CT

C G

C GT T

T

C

T G A G TA G

C T

T C T

G

G A

T T

A T T C

A A A

C G

A

T

T C

G A

C

C G C T A C A

C

C T

T T G

C C T

C C

A G T A

T T

C

T G

T

A

G A C C C

T C

T

G G

A T T T

A T

C A A

T

T

A G C

T A G C

T

A T C T G G T

G A

T

C

C

A

T

C A A TACT

T

T T

T A A T T G

T A

T G G

A A C G G

C

G

C C A ATCTT

C G T

C

A

T

A C

T T G G T

C G T T

C

A G C AG

A GT A A C G

T G C

G C

T

G T

GG

TA T GG

C C A A

T T C T

G

G A T C A A

T

A A C

A

G A

A

T T A C C

G A A

G

C C A T A A A

G T

C

C G

A

C C

T

A

T C A T G

G

A TA

G T

A

C AT A G

A

C A C

A GA G T G G

A G C T G

G

T C G G C A T

C G G T G

G T G G T

T T A T

G T

T C A

C A

G

A G

C T A

A

A

A G C C

T

T

A

G

T T G G

GG

A

A A T

T

A

T C

G

G T

A A

A C

A C C

A	C

T

A

C T

AC A G T G

G G T A

T

C T

T G

A

C C C C

A

C GA

G T

C

T A A

C T A

T G	T

C

T C C

T G

G

A T

T

*rs11556887* RNA secondary structure

*rs7580900* RNA secondary structure

*rs6436915* RNA secondary *structure*

*rs7580912* RNA secondary structure

G T G G	T

T	C

T A T

C

C G T A C G

T A G C T A

G T

A T

A T

AC

C

G

C A G T A A

G

G

A

T

G G G T A A G T A

C

C

C

T

A

AGC

A C

G T C C C T GC

G

G T A T

A

T G

TT

A T C G

C C C A

A G

C

G G

A

A TC

A A T G

T

T

A

C A C T GG

T G

G G T

T

A

T G T T

A G A TAA	T C

GC T A TA C

G

G T

A

C C	G

A

T T T

C C A

C

A

C

G T CC

T G

A

C G A A T

G

G T

G

G C

G CA

C G

T

T

T T C

A G

G

A

A

C A G T T G

A

A T

C

A

A

G A

T G T T

G

C

G

G

G

T

C

T

A

G G T

A

A

A

T

T A C G T G

G C A T

A	C

C G T A T

GT A

G CA TTCG

A

G

T

A

T

G C CTTGTC

C

A

G T

G T

T A

A

A

A T A C

A G

G T C G A T

A T

A T T A T

G C G C C A

C G C G

G

C

C A C A G G A

TA

T

C

T A

A

T T A A C

T

C T T G A C

T

T T T T

C

C G T T A

C C A G C G

G C

G CTG

T AT

T A C G

C GT

C

A T C G

A

C GG

A

A

G A T

T

G A G CA

C G

A

A CG	G

T

GC A

TC G

C G

A G G

G T

AC	G

T A

G C

T A C T

C C

A G

G

G

G A

T A A

T A

G T

G T C G

A

G A TA

C G

A

T

G T A C G

A

T A

T A C G T A C G C G C G

G	G

T ACT

AC G

T ATG

T

C G A AC G

C G A T G C T A

A

GC G CT G

T A

A G GC G

T

A

T

C

C AG

C C

CC G

A T

G T

T A C G

A A

G C

T A

T

G T T A

**T**

T A A

C GA A A T A

G T GG

C G

A A G G

C

A A C G A

A T G C T A

C GT T G

A T

G T

T A

G C

G CG T T

A T

C

G C C G

G C G T T A A

T

CG C

G T

A T A T

T

G C G T

G T

T A

T G C G A T

T

T

T

T A T C G

AC G

C

T

T T C

Rs9061 secondary RNA secondary structure

***Supplementary Figure 1: In silico predicted SP110 SNP-induced RNA secondary structures.*** *The structures represent secondary RNA conformations resulting in minimum free energy (MFE) release.*
