# Supplementary material for: Integrated in silico assessment of the regulatory and structural consequences of pulmonary tuberculosis-associated SP110 polymorphisms
Source: Sci Rep. 2026 Aug 6;16:36800. doi: 10.1038/s41598-026-65881-y (PMC13736213; doi:10.1038/s41598-026-65881-y)
Supplement: Supplementary file 2 [file 41598_2026_65881_MOESM2_ESM.docx]

***Supplementary Table S1: Impact of single nucleotide variants on RNA secondary structure stability***

| **rsID** | **Reference MFE**  **structure**  **(kcal/mol)** | **Variant MFE**  **structure**  **(kcal/mol)** | **\|Δ MFE\|**  **(kcal/mol)** | **Reference Centroid**  **structure**  **(kcal/mol)** | **Variant Centroid**  **structure**  **(kcal/mol)** | **\|Δ Centroid\|**  **(kcal/mol)** |
| --- | --- | --- | --- | --- | --- | --- |
| rs11556887 | -109.10 | -109.10 | 0.00 | -58.38 | -64.77 | 6.39 |
| rs1346311 | -87.40 | -86.00 | 1.40 | -82.20 | -79.50 | 2.70 |
| rs1427294 | -84.30 | -84.40 | 0.10 | -59.94 | -60.04 | 0.10 |
| rs2114592 | -95.50 | -95.50 | 0.00 | -83.40 | -82.90 | 0.50 |
| rs2241525 | -92.30 | -91.60 | 0.70 | -47.60 | -73.37 | 25.77 |
| rs3948464 | -64.70 | -67.20 | 2.50 | -49.81 | -59.58 | 9.77 |
| rs6436915 | -85.90 | -81.20 | 4.70 | -58.50 | -10.80 | 47.70 |
| rs7580900 | -100.10 | -101.30 | 1.20 | -79.60 | -80.80 | 1.20 |
| rs7580912 | -92.90 | -91.50 | 1.40 | -66.40 | -65.00 | 1.40 |
| rs9061 | -95.70 | -95.20 | 0.50 | -92.70 | -86.57 | 6.13 |
